# Adenosine A_2A_ receptor antagonist istradefylline reduces daily OFF time in Parkinson’s disease

**DOI:** 10.1002/mds.25418

**Published:** 2013-03-11

**Authors:** Yoshikuni Mizuno, Tomoyoshi Kondo

**Affiliations:** 1 Kitasato University School of MedicineKanagawa, Japan; 2 Rehabilitation Hananoie HospitalTochigi, Japan

**Keywords:** Parkinson’s disease, istradefylline, levodopa, motor complications, wearing-off

## Abstract

**Background**We evaluated the efficacy and safety of istradefylline, a selective adenosine A_2A_ receptor antagonist administered as adjunctive treatment to levodopa for 12 weeks in a double-blind manner in Parkinson’s disease patients with motor complications in Japan.

**Methods**A total of 373 subjects were randomized to receive placebo (n = 126), istradefylline 20 mg/day (n = 123), or istradefylline 40 mg/day (n = 124). The primary efficacy variable was the change in daily OFF time. Other secondary variables were also evaluated.

**Results**The change in daily OFF time was significantly reduced in the istradefylline 20 mg/day (−0.99 hours, *P* = .003) and istradefylline 40 mg/day (−0.96 hours, *P* = .003) groups compared with the placebo group (−0.23 hours). The most common adverse event was dyskinesia (placebo, 4.0%; istradefylline 20 mg/day, 13.0%; istradefylline 40 mg/day, 12.1%).

**Conclusions**Istradefylline reduced daily OFF time and was well tolerated in Japanese PD patients with motor complications on levodopa treatment.

Long-term levodopa treatment causes motor complications such as wearing-off and dyskinesias in Parkinson’s disease (PD).^1^ Various therapeutic approaches have been developed to overcome these difficulties while maintaining adequate therapeutic levodopa levels. However, the complications and dopaminergic side effects of long-term levodopa treatment are not yet fully resolved.^2^ Istradefylline, a selective adenosine A_2A_ receptor antagonist, is considered nondopaminergic because of the lack of effects on dopamine receptors and dopamine-metabolizing enzymes. Istradefylline is a new antiparkinsonian drug that can be added as a new treatment option to current PD therapy.^3^,^4^ In experimental parkinsonian animals, istradefylline, when used in combination with levodopa, exhibits an additive effect on motor control without worsening levodopa-induced dyskinesia.^6^–^9^ All^10^–^14^ but 1^15^ of the previous studies showed a significant decrease in OFF time. A phase 2b Japanese study showed reduction in daily OFF time^16^; it is the purpose of this study to confirm our previous results.

## Patients and Methods

The diagnosis of PD depended on the UK Parkinson’s Disease Society Brain Bank criteria.^17^ Key inclusion criteria included at least 3 doses of levodopa/decarboxylase inhibitor per day (daily dosage of 300 mg), a stable regimen of all antiparkinsonian drugs for at least 4 weeks prior to randomization, at least 2 hours of OFF time per day, and stages 2 to 4 on the modified Hoehn & Yahr stage (OFF state). Key exclusion criteria included a history of neurosurgery for PD, transcranial magnetic stimulation for PD within 6 months before randomization, dementia or a score of 23 or less on the Mini–Mental State Examination (MMSE), pregnant or lactating women, women planning to have children, and prior istradefylline exposure. Subjects were at least 20 years old when written informed consent was obtained.

This study was a multicenter, placebo-controlled, randomized, double-blind, parallel-group, and confirmatory study conducted between July 2009 and February 2011 at 44 investigative Japanese sites with institutional review board approval based on the principles described in the Declaration of Helsinki. After giving informed consent, subjects were randomly assigned to receive istradefylline 20 or 40 mg/day or placebo orally once daily for 12 weeks. Subjects completed diaries for 7 consecutive days before visits in weeks 2, 4, 8, and 12. At each visit, physicians assessed subjects on the Unified Parkinson’s Disease Rating Scale (UPDRS) Parts I–IV and treatment-emergent adverse events (TEAEs). Electrocardiograms (ECGs) were done every 4 weeks. At the end point, the modified Hoehn & Yahr stage and Clinical Global Impression–Improvement of illness (CGI-I) were assessed. Antiparkinsonian drugs were not changed during the 4 weeks prior to randomization. Reductions in the dosages of antiparkinsonian drugs were permitted only for TEAEs.

The primary efficacy variable was the difference between the total hours of awake time per day spent in the OFF state (daily OFF time) from the baseline diaries and those collected at the last visit. The secondary efficacy variables included change in percentage of awake time per day spent in the OFF state, change in total hours of awake time per day spent in the ON state, change in percentage of awake time per day spent in the ON state, and changes in UPDRS Parts I–IV, CGI-I, and the modified Hoehn & Yahr stage. Safety assessments were the same as in the previous study.^16^

All efficacy analyses were conducted on the full analysis set (FAS), defined as subjects who received at least 1 dose of the study drug and submitted at least 4 valid diaries for evaluation for any assessment times after starting the study drug. The statistical methods used were essentially the same as in the previous study.^16^ This study (6002-009) was registered as ClinicalTrials.gov number NCT00955526.

## Results

A total of 373 subjects were randomized as shown in Supporting Fig. 1. A total of 109 placebo, 111 istradefylline 20 mg/day, and 115 istradefylline 40 mg/day subjects completed 12 weeks of treatment. The numbers of patients who were prematurely withdrawn from the study are also shown in Supporting Figure 1. The FAS included 366 subjects (placebo, 123; istradefylline 20 mg/day, 120; and istradefylline 40 mg/day, 123); 7 subjects were excluded from the FAS because of missing 4 valid diaries. The Safety Set included all 373 randomized subjects. Fewer men were included in the istradefylline 20 mg/day group; more subjects used concomitant selegiline in the istradefylline 40 mg/day group; and fewer subjects used concomitant entacapone in the placebo group. Other demographics and characteristics were comparable (Table 1).

**Table 1:** Demographic and baseline characteristics (full analysis set)

Characteristic	Placebo (n = 123)	Istradefylline 20 mg/day (n = 120)	Istradefylline 40 mg/day (n = 123)
Age (y), mean (SD)	65.8 (8.6)	66.1 (8.6)	65.7 (9.0)
Male, n (%)	58 (47.2%)	40 (33.3%)	64 (52.0%)
BMI, mean (SD), kg/m^2^	22.17 (3.59)	22.34 (3.40)	22.37 (3.65)
Time since diagnosis (y), mean (SD)	7.990 (4.453)	7.301 (4.206)	7.730 (4.547)
Time since onset of motor complications (y), mean (SD)	3.432 (3.470)	3.183 (2.759)	3.258 (3.009)
Daily OFF time			
Mean (SD), h	6.31 (2.47)	6.55 (2.72)	5.97 (2.45)
Mean (SD), %	38.91 (14.80)	40.59 (16.19)	36.92 (15.10)
Daily ON time			
Without dyskinesia (h), mean (SD)	8.53 (2.84)	7.93(3.38)	8.50(3.54)
With dyskinesia (h), mean (SD)	1.35(2.50)	1.57(2.75)	1.83(3.30)
With nontroublesome dyskinesia (h), mean (SD)	0.94 (1.95)	1.00 (1.71)	1.13(2.03)
With troublesome dyskinesia (h), mean (SD)	0.41 (1.11)	0.58 (1.63)	0.69 (1.75)
Without troublesome dyskinesia (h), mean (SD)	9.47 (2.54)	8.93 (2.86)	9.64 (2.82)
UPDRS Part I subscale score, mean (SD)	1.1 (1.5)	1.2 (1.4)	1.0 (1.4)
UPDRS Part II subscale score (ON state), mean (SD)	5.9 (5.2)	5.3 (5.2)	5.3 (5.0)
UPDRS Part II subscale score (OFF state), mean (SD)	14.7 (7.7)	14.9 (7.5)	15.4 (8.1)
UPDRS Part III subscale score (ON state), mean (SD)	21.6 (11.6)	21.3 (10.8)	20.7 (11.0)
UPDRS Part IV subscale score, mean (SD)	4.7 (2.0)	5.1 (2.2)	5.0 (2.4)
Daily dosage of prior levodopa (mg), mean (SD)	425.4 (146.4)	430.8 (156.5)	420.5 (131.8)
Concomitant antiparkinsonian medications, n (%)			
Dopamine agonists	112 (91.1%)	103 (85.8%)	103(83.7%)
Anticholinergic agents	20 (16.3%)	12 (10.0%)	19(15.4%)
Selegiline	57 (46.3%)	52 (43.3%)	75 (61.0%)
Entacapone	52 (42.3%)	63 (52.5%)	68(55.3%)
Amantadine	49 (39.8%)	41 (34.2%)	44 (35.8%)
Zonisamide	17 (13.8%)	13 (10.8%)	20 (16.3%)

The changes from baseline at the end point for daily OFF time for placebo, istradefylline 20 mg/day, and istradefylline 40 mg/day were −0.23, −0.99 (*P* = .003), and −0.96 (*P* = .003) hours, respectively. The differences from the placebo were significant, but there were no difference between the 2 groups. The changes for secondary efficacy variable are shown in Table 2; daily ON time without troublesome dyskinesia for placebo, istradefylline 20 mg/day, and istradefylline 40 mg/day were 0.26, 1.09 (*P* = .003), and 1.08 (*P* = .004) hours, respectively. Neither istradefylline 20 mg/day nor istradefylline 40 mg/day increased daily ON time with troublesome dyskinesia. The changes from baseline at end point for UPDRS Part II score (OFF state) for placebo, istradefylline 20 mg/day, and istradefylline 40 mg/day were −0.6, −1.4 (*P* = .034), and −1.7 (*P* = .009), respectively. The changes from baseline at end point for UPDRS Part III score (ON state) for placebo, istradefylline 20 mg/day, and istradefylline 40 mg/day were −2.8, −3.7 (*P* = .086), and 4.9 (*P* = .001), respectively, showing that istradefylline 40 mg/day significantly reduced UPDRS Part III score (Table 2). The percentages of subjects who were “Much improved” plus “Very much improved” at end point for placebo, istradefylline 20 mg/day, and istradefylline 40 mg/day were 10.7%, 20.8% (*P* = .005), and 28.7% (*P* < .001), respectively (Supporting Fig. 2). No differences were observed among the groups for other secondary efficacy variables. Clinical variables such as age, sex, and others did not affect the effectiveness of istradefylline in reducing daily OFF time.

**Table:** Daily OFF/ON time and UPDRS subscale score—actual data and change from baseline values (full analysis set)

	Placebo (n =123)			Istradefylline 20 mg/day (n =120)					Istradefylline 40 mg/day (n =123)						
	Actual	Change	Actual	Change	Actual	Change
**Daily OFF time**						
End point, LS mean (h)	6.05	−0.23	5.29	−0.99	5.31	−0.96
LS mean vs. placebo (*P*)	—	—	—	−0.76 (.003^a^)	—	−0.74 (.003^a^)
End point, LS mean, %	37.24	−1.55	32.24	−6.55	32.62	−6.17
LS mean vs. placebo (*P*)	—	—	—	−4.99 (.002^a^)	—	−4.61 (.003^a^)
**Daily ON time**						
Without dyskinesia						
End point, LS mean (h)	8.60	0.28	9.22	0.9	9.18	0.85
LS mean vs. placebo (*P*)	—	—	—	0.61 (—)	—	0.57 (.033 NS)
With dyskinesia						
End point, LS mean (h)	1.51	−0.08	1.81	0.22	1.68	0.09
LS mean vs. placebo (*P*)	—	—	—	0.30 (—)	—	0.17 (.139 NS)
**With nontroublesome dyskinesia**						
End point, LS mean (h)	0.98	−0.04	1.27	0.25	1.19	0.16
LS mean vs. placebo (*P*)	—	—	—	0.29 (—)	—	0.21 (.108 NS)
**With troublesome dyskinesia**	0.50	−0.06	0.55	−0.01	0.54	−0.02
End point, LS mean (h)	—	—	—	0.05 (—)	—	0.04 (.421 NS)
LS mean vs. placebo (*P*)						
**Without troublesome dyskinesia**						
End point, LS mean (h)	9.61	0.26	10.44	1.09	10.42	1.08
LS mean vs. placebo (*P*)	—	—	—	0.83 (.003^a^)	—	0.81 (.004^a^)
**UPDRS Part I**						
End point, LS mean	0.9	−0.2	1.0	−0.1	1.0	−0.1
LS mean vs. placebo (*P*)	—	—	—	0.1 (—)	—	0.1 (.906 NS)
**UPDRS Part II (ON state)**						
End point, LS mean	5.2	−0.3	5.2	−0.3	5.0	−0.5
LS mean vs. placebo (*P*)	—	—	—	0.0 (—)	—	−0.2 (.290 NS)
**UPDRS Part II (OFF state)**						
End point, LS mean	14.4	−0.6	13.6	−1.4	13.4	−1.7
LS mean vs. placebo (*P*)	—	—	—	−0.8 (.034 NS)	—	−0.1 (.009^a^)
**UPDRS Part III (ON state)**						
End point, LS mean	18.4	−2.8	17.5	−3.7	16.3	−4.9
LS mean vs. placebo (*P*)				−0.9 (.086 NS)	—	−2.0 (.001^a^)
**UPDRS Part IV**						
End point, LS mean	4.7	−0.2	4.8	−0.2	4.5	−0.4
LS mean vs. placebo (*P*)	—	—	—	0.1 (—)	—	−0.2 (.213 NS)

a *P* < .025 (*P* value by Williams test).

Least squares (LS) mean and *P* values are based on the main effects ANCOVA with terms for baseline, investigator and treatment.

NS, not significant.

TEAEs occurred in 51.6%, 65.0%, and 59.7% of subjects in the placebo, istradefylline 20 mg/day, and istradefylline 40 mg/day groups, respectively. Dyskinesia was the most frequently reported TEAE in both istradefylline groups. The other TEAEs are shown in Supporting Table 1. One subject treated with placebo died on day 19. The situation was unknown at the time of death. Serious adverse events were observed in 2 subjects receiving placebo (2 events; toxicity to various agents and breast cancer in situ), in 6 subjects receiving istradefylline 20 mg/day (8 events; pneumonia bacterial, gait disturbance, radius fracture, neuralgia, sciatica, parkinsonism, delirium, and bile duct cancer), and in 6 subjects receiving istradefylline 40 mg/day (6 events; gastric ulcer, bronchitis, myocardial infarction, pneumonia aspiration, hallucination, and rectal cancer). Of these events, gait disturbance, parkinsonism, gastric ulcer, myocardial infarction, and hallucination were considered drug-related TEAEs. All events resolved or were alleviated. No clinically meaningful changes from baseline were observed in laboratory results, body weight, vital signs, ECG, or MMSE score.

## Discussion

Adenosine A_2A_ receptors in striatum are selectively localized on GABAergic output neurons of the striatopallidal pathway.^3^ Increase in GABA release from the globus pallidus after 6-hydroxydopamine injection into the medial forebrain bundle was reversed by systemic injection with istradefylline in rats.^18^ This study (6002-009) demonstrated the efficacy and safety of istradefylline in Japanese PD subjects. Istradefylline 20 and 40 mg/day significantly reduced daily OFF time with increases in daily ON time without troublesome dyskinesia compared with placebo. But no dose response was seen between 20 and 40 mg/day. This was probably because adenosine receptor occupancy in PET was reported to be more than 90% with a dose of adenosine 5 mg in healthy controls.^19^ These decreases in OFF time were independent of subject demographics. Istradefylline 40 mg/day significantly improved UPDRS Part II (OFF state) and Part III (ON state) scores compared with placebo.

The most frequently reported TEAE was dyskinesia, which occurred with a higher incidence in subject treated with istradefylline than with placebo. All occurrences were mild or moderate in severity and were not dose dependent. Findings in this phase 3 study (6002-009) were consistent with the phase 2b study (6002-0608). These findings demonstrate that istradefylline administered as adjunctive therapy to levodopa reduced daily OFF time and further improved motor functions, thus showing a nondopaminergic drug that can be added to any existing PD therapy.

To date, 4 confirmatory studies have been conducted in advanced PD in the United States using a design similar to this study. Three studies reported reduction in daily OFF time,^12^,^13^ but in 1 study, istradefylline did not provide significant improvement,^15^ possibly because of substantial placebo effect. Dyskinesia was the most frequent adverse event in all studies, with a lower incidence in Japan. The efficacy and safety data in this study indicate that oral istradefylline 20 or 40 mg once daily is effective in relieving wearing-off phenomena and further improving motor functions in Japanese PD patients. Therefore, istradefylline can be added on to other existing antiparkinsonian therapies.
