# Supplementary material for: Adenosine A2A receptor antagonist istradefylline reduces daily OFF time in Parkinson’s disease
Source: Mov Disord. 2013 Mar 11;28(8):1138–41. doi: 10.1002/mds.25418 (PMC3842830; doi:10.1002/mds.25418)
Supplement: Supplementary file 1 [file mds0028-1138-sd1.doc]

Supplementary Figure 1 Subject flow diagram.

Supplementary Figure 2 Clinical Global Impression - global improvement at endpoint (full analysis set)
