# Supplementary material for: Adenosine A2A receptor antagonist istradefylline reduces daily OFF time in Parkinson’s disease
Source: Mov Disord. 2013 Mar 11;28(8):1138–41. doi: 10.1002/mds.25418 (PMC3842830; doi:10.1002/mds.25418)
Supplement: Supplementary file 2 [file mds0028-1138-sd2.doc]

| Supplementary Table 1 Treatment emergent adverse events reported in 3% or more of subjects | | | | | | |
| --- | --- | --- | --- | --- | --- | --- |
|  | Placebo | | Istradefylline  20 mg/day | | Istradefylline  40 mg/day | |
|  | (N=126) | | (N=123) | | (N=124) | |
|  | n | (%) | n | (%) | n | (%) |
| Subjects with any TEAE | 65 | 51.6 | 80 | 65.0 | 74 | 59.7 |
| Dyskinesia | 5 | 4.0 | 16 | 13.0 | 15 | 12.1 |
| Nasopharyngitis | 11 | 8.7 | 10 | 8.1 | 7 | 5.6 |
| Constipation | 3 | 2.4 | 7 | 5.7 | 4 | 3.2 |
| Somnolence | 4 | 3.2 | 8 | 6.5 | 2 | 1.6 |
| Blood creatine kinase increased | 7 | 5.6 | 4 | 3.3 | 1 | 0.8 |
| Nausea | 4 | 3.2 | 2 | 1.6 | 5 | 4.0 |
| [Back pain](javascript:detail('LLT','10003988');) | 3 | 2.4 | 5 | 4.1 | 3 | 2.4 |
| Contusion | 2 | 1.6 | 4 | 3.3 | 2 | 1.6 |
| Orthostatic hypotension | 2 | 1.6 | 4 | 3.3 | 1 | 0.8 |
| Hallucination, visual | 0 |  | 1 | 0.8 | 5 | 4.0 |
| Blood glucose increased | 1 | 0.8 | 1 | 0.8 | 4 | 3.2 |
| Spinal compression fracture | 1 | 0.8 | 4 | 3.3 | 1 | 0.8 |
