# Supplementary material for: Adenosine A2A receptor antagonist istradefylline reduces daily OFF time in Parkinson’s disease
Source: Mov Disord. 2013 Mar 11;28(8):1138–41. doi: 10.1002/mds.25418 (PMC3842830; doi:10.1002/mds.25418)
Supplement: Supplementary file 3 [file mds0028-1138-sd3.doc]

**APPENDIX**

The Japanese Istradefylline Study Group Investigators included the following members: S Hisahara (Sapporo Medical University Hospital); M Baba (Aomori Prefectural Central Hospital); T Abe (Abe Neurology Clinic); T Maeda (Research Institute for Brain and Blood Vessels Akita); A Takeda (Tohoku University Hospital); M Suzuki (Sendai East Neurosurgical Hospital); R Koike (Nishi-Niigata Chuo National Hospital); K Hirata (Dokkyo Medical University Hospital); A Tamaoka (Tsukuba University Hospital); K Okamoto (Gunma University Hospital); H Morita (Shinshu University Hospital); M Asahina (Chiba University Hospital); S Nogawa (Tokyo Dental College Ichikawa General Hospital); H Shimura (Juntendo University Urayasu Hospital); N Suzuki (Keio University Hospital); H Utsumi (Tokyo Medical University Hospital); Y Shimo (Juntendo University Hospital); R Hanajima (The University of Tokyo Hospital); M Murata (National Center of Neurology and Psychiatry); F Yokochi (Tokyo Metropolitan Tama Medical Center); M Yokochi (Ebara Hospital); S Nakamura (Juntendo Tokyo Koto Geriatric Medical Center); K Hasegawa (National Hospital Organization Sagamihara National Hospital); H Takahashi (Tokai University Hospital); N Kawashima (Kawashima Neurology Clinic); S Ikebe (Ikebe Clinic); T Ohashi (Seirei Hamamatsu General Hospital); T Atsumi (Atsumi Neurology Clinic); T Hattori (Honmachi Clinic); H Sawada (National Hospital Organization Utano Hospital); Y Tatsuoka (Tatsuoka Neurology Clinic); S Matsumoto (Kitano Hospital); H Fujimura (Toneyama National Hospital); K Toda (Toda Internal medicine & Rehabilitation Clinic); H Miwa (Wakayama Medical University Hospital); M Yamamoto (Kagawa Prefectural Central Hospital); M Nagai (Ehime University Hospital); T Yuhi (University of Occupational and Environmental Health, School of Medicine Hospital); T Yamada (Fukuoka University Hospital); S Kuno (Kyoto Shijo Hospital); H Mori (Kurashiki Central Hospital); H Mochizuki (Kitasato University Hospital); J Yoshinaga (Yoshinaga Neurology Clinic); and K Uekawa (National Hospital Organization Kumamoto Minami Hospital).
